# Thoracic Paravertebral Block with Adjuvant Dexmedetomidine in Video-Assisted Thoracoscopic Surgery: A Randomized, Double-Blind Study

**DOI:** 10.3390/jcm8030352

**Published:** 2019-03-12

**Authors:** Boohwi Hong, ChaeSeong Lim, Hyemin Kang, Hongsik Eom, Yeojung Kim, Hyun Jin Cho, Woosik Han, Sunyeul Lee, Woosuk Chung, Yoon-Hee Kim

**Affiliations:** 1 Department of Anesthesiology and Pain Medicine, Chungnam National University Hospital, 282 Munhwa-ro, Jung-gu, Daejeon 35015, Korea; koho0127@gmail.com (B.H.); limtwo2@gmail.com (C.L.); man4ok2017@naver.com (H.K.); tdoreins@naver.com (H.E.); yeojung80@naver.com (Y.K.); neoquack@naver.com (S.L.); woosuk119@gmail.com (W.C.); 2 Department of Anesthesiology and Pain Medicine, College of Medicine, Chungnam National University, 266 Munhwa-ro, Jung-gu, Daejeon 35015, Korea; 3 Department of Thoracic & Cardiovascular Surgery, Chungnam National University Hospital, 282 Munhwa-ro, Jung-gu, Daejeon 35015, Korea; irainy79@naver.com (H.J.C.); innomi79@gmail.com (W.H.)

**Keywords:** dexmedetomidine, video-assisted thoracoscopic surgery (VATS), thoracic paravertebral block (TPVB), pain, postoperative

## Abstract

Background: The addition of the adjuvant dexmedetomidine to a nerve block improves the quality of the block and reduces perioperative opioid consumption. The aim of this study was to assess the effect of dexmedetomidine as an adjuvant for the thoracic paravertebral block (TPVB) in postoperative pain control after video-assisted thoracoscopic surgery (VATS). Methods: Sixty-six males, aged 15–40 years, with spontaneous pneumothorax scheduled for VATS wedge resection were enrolled. Following surgery, ultrasound-guided TPVB was performed on the T3 and T5 levels with 30 mL of 0.5% ropivacaine, plus adjuvant dexmedetomidine 50 μg or normal saline. The primary outcome was cumulative fentanyl consumption at 24 h. Pain severity, the requirement for additional rescue analgesics, hemodynamic variations, and side effects were also evaluated. Results: Median postoperative cumulative fentanyl consumption at 24 h was significantly lower in the dexmedetomidine group (122.6 (interquartile range (IQR) 94.5–268.0) μg vs. 348.1 (IQR, 192.8–459.2) μg, *p*-value = 0.001) with a Hodges–Lehman median difference between groups of 86.2 (95% confidence interval (CI), 4.2–156.4) mg. Coughing numeric rating scale (NRS) was lower in the dexmedetomidine group at postoperative 2, 4, 8, and 24 h. However, resting NRS differed significantly only after 4 h postoperative. Conclusions: Dexmedetomidine as an adjunct in TPVB provided effective pain relief and significantly reduced opioid requirement in VATS.

## 1. Introduction

Video-assisted thoracic surgery (VATS) has been widely used in the treatment of spontaneous pneumothorax. Minimally invasive surgery is more effective than open thoracotomy in reducing postoperative pain and complications, as well as shortening operation time and hospital stay [1]. However, the management of postoperative pain—especially early postoperative pain—remains a concern of many anesthesiologists and thoracic surgeons [2,3]. 

Thoracic paravertebral block (TPVB) may reduce postoperative opioid consumption and provide effective pain control [4,5,6]. The effect of TPVB on pain relief is similar to that of epidural analgesia, which is considered the gold standard [7,8], and the development of TPVB techniques using ultrasound has made it easier and more accessible [9]. However, the duration of the local anesthetic itself, such as ropivacaine, is limited to 8–14 h and the rebound pain after the nerve block wears off diminishes the analgesic benefit of the nerve block [10]. Dexmedetomidine, an α2 adrenergic receptor agonist developed as a short-term sedative [11], has shown sympatholytic, analgesic, and opioid-sparing effects, whilst adjuvant dexmedetomidine has been found to improve the duration and quality of nerve block [12,13,14].

It remains unclear whether TPVB reduces opioid consumption in VATS surgery [2,5,15,16]. Therefore, the present study investigated the effect of dexmedetomidine on the opioid-sparing properties of TPVB after VATS surgery.

## 2. Materials and Methods

The study protocol was approved by the Chungnam National University Hospital Institutional Review Board (IRB CNUH 2015-11-003-004), and the trial was registered at the Clinical Research Information Service, a clinical trial registry in Korea (KCT0001770). This parallel group, randomized, controlled, double-blind study enrolled patients scheduled for wedge resection under VATS at Chungnam National University Hospital (Daejeon, Korea), with all patients providing written informed consent.

All patients were males aged 15–40 years with American Society of Anesthesiologists physical status I or II scheduled for wedge resection under VATS. Patients were excluded if they refused TPVB, had a coagulopathy or bleeding disorder, were being treated with an antiplatelet agent, had a local infection at the injection site, were hypersensitive to local amide anesthetics, or were hypersensitive or allergic to dexmedetomidine. Patients were also excluded if they had central neuropathy, a body mass index >35 kg/m^2^, uncontrolled diabetes mellitus, significant cardiopulmonary disease, or psychiatric disease.

After induction of general anesthesia, the study subjects were randomized 1:1 to the dexmedetomidine (30 mL of 0.5% ropivacaine plus 0.5 mL of 50 µg dexmedetomidine) group or control (30 mL of 0.5% ropivacaine plus 0.5 mL of normal saline) group using a computer-generated random number table with a block size of four. To conceal group allocation, the random number table was saved in Redcap software, which was used for randomization and data management. This randomization function in Redcap was accessible only to researchers preparing the study drugs; these researchers were not involved in patient monitoring or outcome analyses. The study drugs were prepared in a space other than the operating room in which surgery was performed.

On the day before surgery, a professional nurse inserted 18-gauge intravenous catheters into each patient’s forearm, and the researcher explained the pain numeric rating scale (NRS) to the patient. Patients were premedicated with anticholinergics (0.04 mg/kg of glycopyrrolate). Routine monitoring included electrocardiography, pulse oximetry, and noninvasive blood pressure measurements. Patients were anesthetized by standard methods using propofol, remifentanil, rocuronium, and sevoflurane. Patients were intubated with a 37 or 39 Fr double-lumen tube, with the optimal position of the tube and one-lung ventilation confirmed by bronchoscopy. Bronchoscopy was again performed after the patient was changed from the supine to the lateral decubitus position. One-lung ventilation was adjusted to 5–6 mL/kg of ideal body weight, and the tidal volume and respiration rate were adjusted to 35–40 mmHg of end-tidal CO_2_. Surgery was performed using either a single port or 2–3 ports, depending on the difficulty of surgery and the position of the lesion. At the end of the operation, a chest tube was inserted into the main port and lung expansion was confirmed directly by thoracoscopy, followed by a recruitment maneuver at 30 mmHg.

### 2.1. Thoracic Paravertebral Block

During the completion of wound dressing immediately after surgery, the lateral decubitus position was maintained with two-lung ventilation under general anesthesia. All blocks were performed by a single researcher who was blinded to the group assignment and study drug. A 22 gauge, 80 mm, echogenic needle (SonoPlex cannulas, Pajunk^®^, Geisingen, Germany), MylabTM25 Gold (Esaote, Genova, Italy), and a linear probe (LA435: 6-18 MHz, Esaote, Genova, Italy) were used. Povidone-iodine was used to make an aseptic field. The locations of the spinous process, transverse process, and ribs were confirmed by ultrasound. The first rib was identified in the parasagittal plane, and the ribs were counted in order. The T3–4 intercostal space and the transverse process were confirmed by an in-plane intercostal approach as described by Yasuyuki Shibata [17]. The needle was located in the thoracic paravertebral space and 1 mL of test dose was applied to confirm the displacement of the pleura. The assistant subsequently injected 14 mL of prepared drug. The same procedure was applied to the fifth thoracic paravertebral space.

After two injections, the patient’s position was changed to a supine position. The inhalation anesthetic was discontinued and muscle relaxation was reversed by pyridostigmine and glycopyrrolate. When the patient recovered consciousness and spontaneous breathing was sufficient, the endotracheal tube was removed. The level of mental status was monitored in the post-anesthesia care unit (PACU), taking into account the time that the drug had sufficiently spread. Successful TPVB was assessed around the level of the nipple by a pinprick test with a stylet of 22-gauge spinal needle 30 min after the block. During this assessment, the pain score was again explained to the patient and analgesics were administered through the patient-controlled analgesia (PCA) device (GemStar^TM^, Hospira, IL, USA) when a patient reported an NRS score ≥4. Patients who experienced bradycardia—defined as a heart rate <50 beats/min—were treated with 0.5 mg of atropine.

To increase the reliability of the data, each PCA device was collected, and the log records stored in the device were transferred to an electronic medical records (EMRs) system. Each PCA device was set to administer a 0.5 μg/kg bolus dose of fentanyl without background infusion, with a lockout time of 15 min and a total allowable fentanyl dose of 1000 μg. If pain control was insufficient with PCA alone, patients were administered 30 mg of ketorolac, followed, if necessary, by 25 mg of pethidine. All patients received intravenous nefopam 20 mg (12-h interval) as part of multimodal analgesia.

The primary outcome was postoperative cumulative fentanyl consumption at 24 h. Secondary outcomes were the use of analgesics in the PACU, resting and coughing NRS over time, and maximum NRS during the 24 h study period.

### 2.2. Statistical Analyses

The required sample size was calculated based on a previous study [4,5] using G*Power (version 3.1, Franz Faul & Edgar Erdfelder, Trier, Germany). In those studies, morphine consumption for 24 h after TPVB for VATS was approximately 30 ± 10 mg, which was converted to the equivalent amount of fentanyl. A study using dexmedetomidine as an adjuvant in TPVB after breast cancer surgery found that the analgesic requirement was reduced by 25% [18]. Assuming a 25% reduction in fentanyl consumption and a power of 80% with a risk of 0.05 for type 1 errors (two-tailed, effect size of 0.75), the minimum number of patients required in each group was 29. Allowing for a 10% dropout rate, the planned number of patients was 66. 

All statistical analyses were performed using R software version 3.4.3 (R Project for Statistical Computing, Vienna, Austria). Normality was tested using the Shapiro–Wilk test. Continuous variables were analyzed by Student’s *t*-test or the Mann–Whitney test, depending on the normality of the data, and recorded as mean ± SD or median (interquartile range (IQR)). Point estimation and the confidence interval of the Hodges–Lehmann’s median were calculated using SAS software (version 9.3 for Windows, SAS Korea, Seoul, Korea). Categorical variables were recorded as numbers (%) and analyzed using the Chi-square test or Fisher’s exact test. A two-tailed *p*-value of <0.05 was considered statistically significant. Repeated measurements were analyzed using repeated measures analysis of variance. If the differences were significant, Bonferroni’s correction was used to reduce the probability of a type 1 error occurring when multiple testing was performed on each point.

## 3. Results 

**Figure 1:**
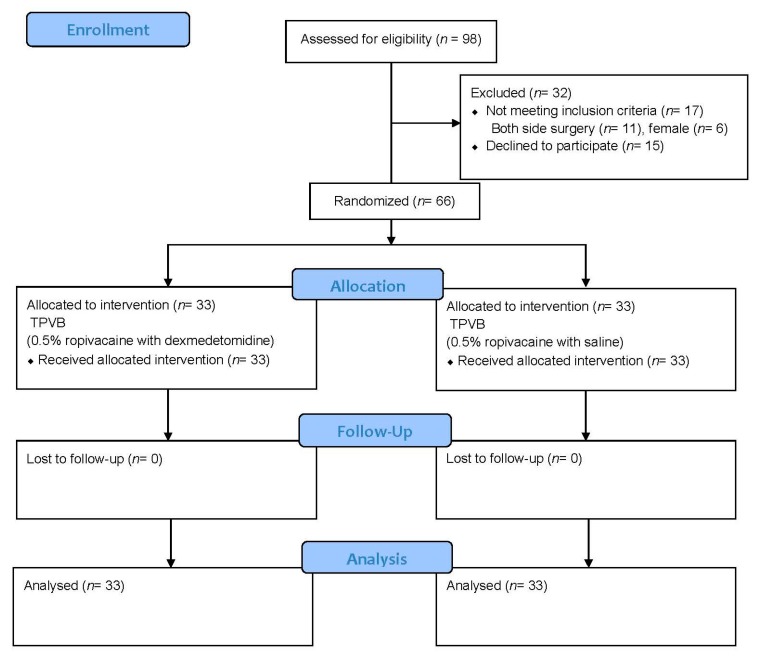
The Consolidated Standards of Reporting Trials (CONSORT) flow diagram of study participants. TPVB: thoracic paravertebral block.

From January 2016 to December 2017, 98 patients were assessed for eligibility, and 32 were excluded. These 32 patients consisted of 11 patients who had surgery on both sides, 6 female patients, and 15 patients who refused study participation. The remaining 66 patients were randomized in a 1:1 ratio: 33 to the dexmedetomidine group and 33 to the control group. None of the participants were lost to follow-up. The Consolidated Standards of Reporting Trials (CONSORT) diagram is shown in Figure 1, followed by a comparison of the demographic and clinical characteristics of the two groups (Table 1).

**Figure 2:**
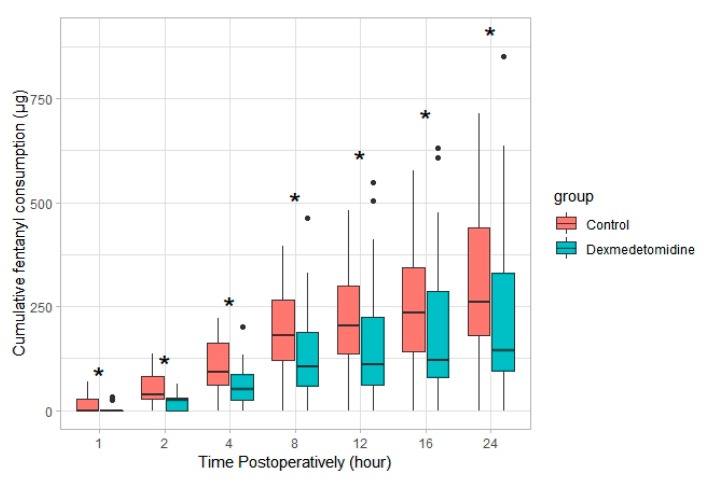
Cumulative fentanyl consumption over time in both groups. Data are expressed as median (interquartile range). * *p* < 0.05. ● outlier (any data point more than 1.5 interquartile ranges below the first quartile or above the third quartile).

**Figure 3:**
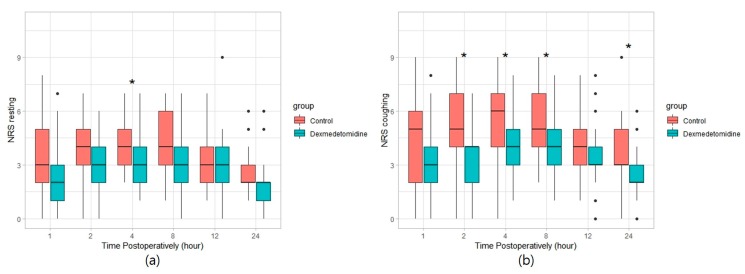
Pain score over time while (**a**) resting and (**b**) coughing. Data are expressed as median (interquartile range). * *p* < 0.05. ● outlier (any data point more than 1.5 interquartile ranges below the first quartile or above the third quartile).

Postoperative cumulative fentanyl consumption at 24 h was significantly lower in the dexmedetomidine group than in the control group (122.6 (IQR, 94.5–268.0) μg vs. 348.1 (IQR, 192.8–459.2) μg, *p* = 0.001), with a Hodges–Lehman median difference of 86.2 (95% confidence interval (CI), 4.2–156.4) mg between groups. The number of patients requiring analgesics in the PACU was significantly lower in the dexmedetomidine group than in the control group (12.1% vs. 42.4%, *p* = 0.013; relative risk (95% CI) = 0.368 (0.15–0.899)). The difference in opioid consumption, which began during the early postoperative period, continued over the entire 24 h postoperative period (Figure 2). NRS pain score for coughing was lower in the dexmedetomidine group at postoperative 2, 4, 8, and 24 h. However, NRS pain score for resting was not significantly different at any point postoperatively, other than at 4 h (Figure 3). Maximum NRS pain scores for both resting and coughing over 24 h were significantly lower in the dexmedetomidine group (Table 2). 

**Figure 4:**
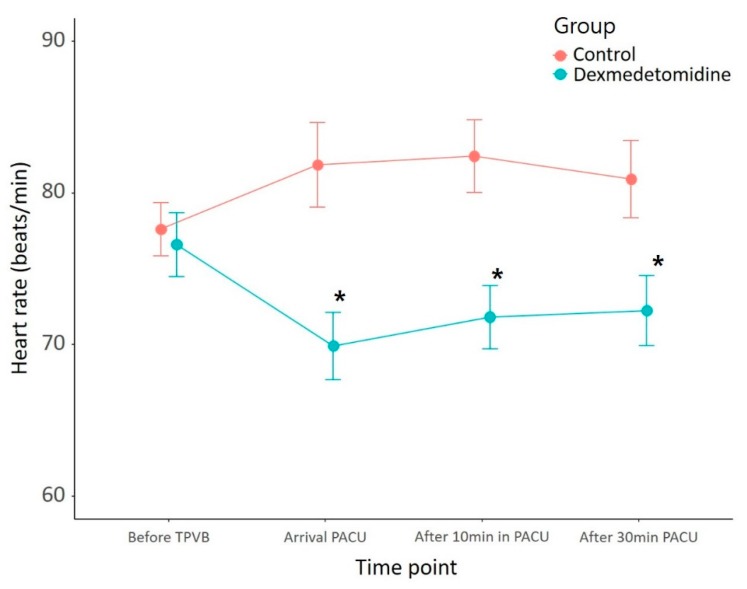
Changes in heart rate over time in the post-anesthesia care unit. The changes over time differed significantly in the two groups (*p* = 0.002). * *p* < 0.013 using Bonferroni’s correction for multiple comparisons. PACU: post-anesthesia care unit; TPVB: thoracic paravertebral block.

The frequency of additional rescue analgesics, length of hospital stay, and postoperative nausea were not different between the two groups (Table 2). Although postoperative heart rate was lower in the dexmedetomidine group, no patient required atropine (Figure 4). 

## 4. Discussion

This study found that dexmedetomidine as an adjuvant for TPVB for postoperative pain control after VATS had superior analgesic efficacy compared to TPVB alone. Effective pain control during the acute phase reduced the use of analgesics for up to 24 h. 

The mechanism of adjuvant dexmedetomidine for nerve block is not fully understood, but it is likely to be multifactorial. The lower pain score in the early postoperative period and reduced opioid consumption up to 24 h postoperatively are thought to be a mixed effect of several mechanisms. Dexmedetomidine has centrally mediated analgesic effects at the cerebral and spinal levels through an α2-receptor mechanism. Its direct binding to α2 adrenoreceptors in the locus coeruleus may explain the extended effects of intravenous dexmedetomidine on nerve block duration [19]. A trial in patients undergoing arthroscopic shoulder surgery showed that systemic dexmedetomidine prolonged interscalene block compared to perineural dexmedetomidine [13]. The peripheral effects of α2 agonists resulting in analgesia are mediated by a reduction in the release of norepinephrine as well as by α2 receptor-independent inhibition of nerve fiber action potentials. According to Fritsch et al. [20], the perineurally injected dexmedetomidine prolonged nerve block for several hours, but blood concentration of dexmedetomidine was very low, so a systemic mechanism is not considered to block prolongation. Also, the bilateral paired study of controlling for systemic effects found that the duration of saphenous nerve block was significantly longer in the adjuvant dexmedetomidine plus ropivacaine [21]. Perineural dexmedetomidine prolonged sensory block of the ulnar nerve by 60%, and systemic dexmedetomidine also prolonged sensory block by 10% compared with placebo [22]. 

Because this study was a parallel group study with ropivacaine alone as the active comparison group, dexmedetomidine was not intravenously injected into patients. Therefore, it is difficult to determine the precise mechanism by which adjuvant dexmedetomidine has opioid-sparing effects in TPVB. Dexmedetomidine has an intrinsic analgesic effect. Therefore, in the absence of a study group administered dexmedetomidine alone (without ropivacaine) or sham block (placebo control), it was not possible to determine whether the observed benefits were due to the intrinsic systemic analgesic effect of dexmedetomidine or to its potentiation of the analgesic effect of the nerve block.

TPVB has been shown to reduce various complications of the epidural block in patients undergoing breast surgery, and is considered the gold standard [23]. However, it is unclear whether opioid consumption is reduced after TPVB in breast surgery, with studies showing that its effect is limited to the early postoperative period, suggesting that it is due to the duration of ropivacaine anesthesia [24,25]. The adjuvant dexmedetomidine, which increases the quality and duration of TPVB, has been consistently reported to reduce postoperative opioid consumption in breast surgery [18,26]. In VATS, it is also not clear whether TPVB reduces opioid consumption. A single TPVB was reported effective in controlling pain for up to 6 h [2], but there was no difference in morphine consumption after 6 h. In addition, a single TPVB did not reduce opioid consumption at 24 and 48 h after VATS surgery [15]. Preoperative multiple-injection TPVB, however, significantly reduced cumulative morphine consumption for 48 h postoperatively [5]. The reason for these conflicting results may have been due to differences in study design, differences in the number of injections, the volume of local anesthetics administered, the number of ports, the patient population, and operation times. Furthermore, nociceptive pathways in thoracic surgery are complex [27]. Nociceptive somatic afferents are conveyed by intercostal nerves after skin incision, rib retraction, muscle splitting, injury to the parietal pleura, and chest drain insertion into the ipsilateral dorsal horn of the spinal cord (T4–T10). Pain signals of the diaphragmatic pleura are transmitted by the phrenic nerve, usually expressed as ipsilateral shoulder pain. Additionally, insertion of the tube can irritate the pleural dome, causing postoperative pain. The vagus nerve running along the pericardium also controls pain of mediastinal pleural origin. As pain after thoracic surgery is caused by multiple pathways, TPVB alone is insufficient for complete pain control, which may explain the differences in results observed in previous studies. 

This study has several limitations. Firstly, all patients in the dexmedetomidine group received 50 µg of dexmedetomidine. As 200 µg of dexmedetomidine was dissolved in 2 mL saline, it was difficult to fine-tune the dose. Instead, using a small volume of dexmedetomidine could have minimal effect on the concentration of local anesthetics. Most studies have used weight-based dosing of dexmedetomidine (1 μg/kg), and similar doses were applied to the lean populations in this study. Another limitation was the lack of a dose–response study, which likely would have shown that higher doses are more effective. However, a previous study found that a high intravenous dose of 2 μg/kg was associated with a greater need of ephedrine for intraoperative hypotension, suggesting a trade-off between the benefits and risks of high-dose adjuvant dexmedetomidine [28]. This study also did not evaluate the dermatomal spread of the blockade representing its analgesic quality. Analgesia and the spread of local anesthetic can be inconsistent. In addition, satisfactory postoperative analgesia may be achieved after the block, although targeted dermatomes are only partially blocked [29]. However, this study tried to eliminate the sparing level by administering a large volume of 15 mL to the third and fifth levels. Another limitation of this study was its inclusion of male patients only. Females have been reported to feel more severe, frequent, and diffuse pain than males with similar disease processes [30]. The differences in pain sensitivity, pain facilitation, and pain inhibition may be due to sex hormones, endogenous opioid function, genetic factors, psychosocial processes such as pain coping and catastrophizing, and gender roles [31]. Also, pneumothorax is much more prevalent in males [32]. By assessing males only, this study was able to reduce any bias resulting from sex differences in pain. 

## 5. Conclusions

Dexmedetomidine as an adjunct in TPVB provided effective pain relief and significantly reduced opioid requirement in VATS.

## Figures and Tables

**Table 1:** Demographic and clinical characteristics of the study patients.

	Dexmedetomidine Group (*n* = 33)	Control Group (*n* = 33)
Age (year)	19.0 (17.0–22.0)	19.0 (18.0–22.0)
Height (cm)	173.3 (169.5–178.2)	173.1 (166.6–178.2)
Weight (kg)	58.2 ± 8.0	57.8 ± 8.1
BMI (kg/m^2^)	19.2 ± 2.2	19.3 ± 2.4
Port number		
1	21 (63.6)	13 (39.4)
2	2 (6.1)	4 (12.1)
3	10 (30.3)	16 (48.5)
Previous VATS history	7 (21.2)	11 (33.3)
Pleural adhesion	3 (9.1)	9 (27.3)

Variables described as median (interquartile range); mean ± standard deviation (SD); or number (%). BMI: body mass index; VATS: video-assisted thoracoscopic surgery.

**Table 2:** Postoperative results in the study patients.

	Dexmedetomidine Group (*n* = 33)	Control Group (*n* = 33)	*p*
Extubation time (min)	11.0 (9.0–13.0)	10.0 (8.0–12.0)	0.071
PACU stay time (min)	44.0 (39.0–53.0)	42.0 (38.0–49.0)	0.517
Analgesic request in PACU	4 (12.1%)	14 (42.4%)	0.013
Maximum NRS pain score			
Resting	4.0 (3.0–5.0)	5.0 (4.0–6.0)	0.032
Coughing	5.0 (4.0–6.0)	7.0 (5.0–7.0)	0.003
Rescue analgesic (ketorolac)	8 (24.2%)	7 (21.2%)	1.000
Rescue analgesic (pethidine)	1 (3.0%)	3 (9.1%)	0.606
Discharge day after operation	3.0 (3.0–5.0)	3.0 (3.0–5.0)	0.455
Nausea	4 (12.1%)	3 (9.1%)	1.000

Data are presented as median (interquartile range) or number (%). NRS: numeric rating scale; PACU: post-anesthesia care unit.
